# H Bonding at the Helix-Bundle Crossing Controls Gating in Kir Potassium Channels

**DOI:** 10.1016/j.neuron.2007.07.026

**Published:** 2007-08-16

**Authors:** Markus Rapedius, Philip W. Fowler, Lijun Shang, Mark S.P. Sansom, Stephen J. Tucker, Thomas Baukrowitz

**Affiliations:** 1 Institute of Physiology II, Friedrich Schiller University, D-07743 Jena, Germany; 2 Structural Bioinformatics and Computational Biochemistry Unit, Department of Biochemistry, University of Oxford, OX1 3QU Oxford, UK; 3 Oxford Centre for Gene Function, Department of Physiology, Anatomy and Genetics, University of Oxford, OX1 3PT Oxford, UK

**Keywords:** MOLNEURO, SIGNALING, PROTEINS

## Abstract

Specific stimuli such as intracellular H^+^ and phosphoinositides (e.g., PIP_2_) gate inwardly rectifying potassium (Kir) channels by controlling the reversible transition between the closed and open states. This gating mechanism underlies many aspects of Kir channel physiology and pathophysiology; however, its structural basis is not well understood. Here, we demonstrate that H^+^ and PIP_2_ use a conserved gating mechanism defined by similar structural changes in the transmembrane (TM) helices and the selectivity filter. Our data support a model in which the gating motion of the TM helices is controlled by an intrasubunit hydrogen bond between TM1 and TM2 at the helix-bundle crossing, and we show that this defines a common gating motif in the Kir channel superfamily. Furthermore, we show that this proposed H-bonding interaction determines Kir channel pH sensitivity, pH and PIP_2_ gating kinetics, as well as a K^+^-dependent inactivation process at the selectivity filter and therefore many of the key regulatory mechanisms of Kir channel physiology.

## Introduction

Inwardly rectifying K^+^ (Kir) channels play a vital role in many diverse physiological processes, including control of the heart rate, setting the activation threshold of neuronal excitability, hormonal secretion, extracellular K^+^ buffering in the brain, and K^+^ secretion in the kidney. These processes are regulated by specific stimuli which cause Kir channels to undergo a reversible transition between the closed and open state. This is known as gating, and examples of stimuli include intracellular H^+^, which induces pore closure in some Kir channels (pH gating) (Schulte and Fakler, 2000), and phosphatidylinositol-(4,5)-bisphosphate (PIP_2_), which induces pore opening in all known eukaryotic Kir channels (PIP_2_ gating) (Bichet et al., 2003).

The regulation of Kir channels by intracellular H^+^ and PIP_2_ underlies many important aspects of Kir physiology as well as pathophysiology (Donaldson et al., 2003, Hebert, 2003; Lin et al., 2006; Lopes et al., 2002). The mechanism of pH gating has been most widely studied in the Kir1.1 (ROMK) channel, which is found in the apical membrane of renal tubular epithelia where it secretes excess K^+^ into the urine. This K^+^ transport pathway is regulated by intracellular pH (pH gating) as well as by the luminal K^+^ concentration (K^+^-dependent inactivation), and both regulatory mechanisms are essential for the renal control of K^+^ homeostasis. This is highlighted by the fact that inherited mutations in Kir1.1 which interfere with this pH-sensing mechanism result in type II Bartter's syndrome, a hypokalaemic disorder (Hebert, 2003; Hebert et al., 2005; Schulte and Fakler, 2000). Furthermore, the pH sensitivity of Kir1.1, and other pH-sensitive Kir channels such as Kir4.1 and heteromeric Kir4.1/Kir5.1, is thought to be important for the chemosensitivity (CO_2_ response) of specific neuronal populations in the brainstem (Su et al., 2006) and in the buffering of extracellular K^+^ concentrations by glial cells in the brain (Neusch et al., 2006).

The physiological significance of PIP_2_ regulation has been demonstrated in studies showing that dynamic changes in phosphoinositide metabolism directly modulate many Kir channels (Baukrowitz and Fakler, 2000; Cho et al., 2005; Kobrinsky et al., 2000) and that mutations which impair PIP_2_ activation cause a variety of channelopathies including Bartter's syndrome, Andersen's syndrome, and congenital hyperinsulinaemia (Donaldson et al., 2003; Lin et al., 2006; Lopes et al., 2002).

Most Kir channels are inhibited by intracellular acidosis; however, their pH sensitivity differs markedly. A key determinant of pH sensitivity is a lysine residue in the first transmembrane domain (TM1) at the helix-bundle crossing. Kir channels with a lysine at this position (Kir1.1, Kir4.1, Kir4.2, and Kir4.1/Kir5.1) show high pH sensitivity. By contrast, channels lacking a lysine at this position (e.g., Kir2.1, Kir3.x, and Kir6.2) are markedly less pH sensitive but can increase their pH sensitivity if a lysine is introduced. Originally, it was thought that this lysine was the pH sensor (Fakler et al., 1996; Schulte and Fakler, 2000; Schulte et al., 1999). However, recent work has shown that this lysine is not the actual titratable H^+^ sensor but instead a critical component of the gating machinery (Leng et al., 2006; Rapedius et al., 2006). Thus, the specific identity of the pH sensor remains unknown.

Mutations throughout the Kir channel have been shown to alter pH sensitivity (Chanchevalap et al., 2000; Schulte and Fakler, 2000; Leng et al., 2006; Rapedius et al., 2006), suggesting that pH inhibition induces a global conformational change. In Kir1.1, pH inhibition is also intimately coupled to an inactivation process that depends on the extracellular K^+^ concentration (K^+^-dependent inactivation). In zero or low concentrations of extracellular K^+^, Kir1.1 rapidly enters an inactivated state after pH inhibition. This inactivation can be prevented by the presence of extracellular K^+^ and other permeant ions (e.g., Rb^+^, Cs^+^) and is thought to represent a collapse of the pore at the selectivity filter (Dahlmann et al., 2004; Schulte et al., 2001), similar to the C-type inactivation seen in many Kv channels (Baukrowitz and Yellen, 1996; Liu et al., 1996; Smith et al., 1996; Starkus et al., 1997; Yellen, 1998). However, the molecular mechanism which underlies the coupling between pH inhibition and K^+^-dependent inactivation is currently unknown.

The activity of all Kir channels is dynamically regulated by PIP_2_, and depletion of PIP_2_ in the membrane leads to a loss of channel activity. The exact PIP_2_ binding site is currently unknown. However, many (mostly basic) residues which affect PIP_2_ activation are found clustered in the cytoplasmic domains of the Kir channel and thought to represent the PIP_2_ binding domain (Baukrowitz et al., 1998; Bichet et al., 2003; Enkvetchakul and Nichols, 2003; Lopes et al., 2002).

Another class of anionic lipids, long chain coenzyme A esters (LC-CoA), also modulate Kir channel activity by binding to a site which overlaps the PIP_2_ binding site (Rapedius et al., 2005; Rohacs et al., 2003; Schulze et al., 2003). In most cases, LC-CoA binding inhibits Kir channel activity by directly competing with PIP_2_ for channel binding (Rapedius et al., 2005). However, in K_ATP_ channels (Kir6.2/SUR1), both LC-CoA and PIP_2_ produce channel activation (Branstrom et al., 2004; Rohacs et al., 2003). This regulation links Kir channel activity to cellular fatty acid metabolism (Shumilina et al., 2006), fatty acid sensing in hypothalamic neurons (Lam et al., 2005), and pancreatic insulin secretion (Branstrom et al., 2004).

It is assumed that ligands that gate Kir channels (e.g., PIP_2_, H^+^, ATP, G proteins) initiate a conformational change in the cytoplasmic domains that is transduced to the TM helices and thereby open or close the pore. Furthermore, pore opening is thought to involve a widening of the pore at the helix bundle crossing (Bichet et al., 2003; Phillips et al., 2003; Sackin et al., 2005; Sadja et al., 2001; Xiao et al., 2003). However, the precise conformational changes underlying these processes are not well understood, especially when compared to current knowledge about the gating mechanisms in other classes of ion channels (e.g., Kv channels and acetylcholine receptors).

In this study, we demonstrate that both pH gating and PIP_2_ gating in Kir channels share a common structural basis. We are also able, for the first time, to directly measure the kinetics of PIP_2_ gating and pH gating, and we propose that these gating kinetics depend on the presence of an intrasubunit hydrogen bond between TM1 and TM2 at the base of the helix bundle crossing. Furthermore, we demonstrate that this gating mechanism is common to all Kir channels and that it not only controls their response to PIP_2_ but also determines their pH sensitivity and response to extracellular K^+^.

## Results

### Measuring the Kinetics of PIP_2_ Gating in Kir Channels

**Figure 1:**
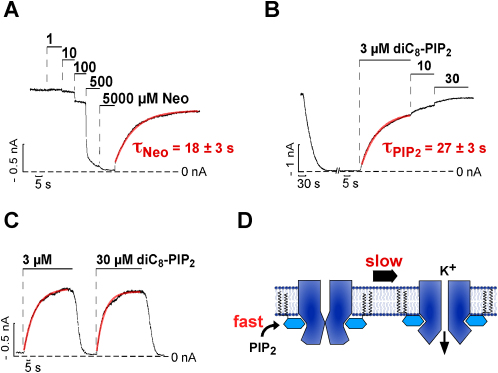
Assay to Measure the Kinetics of PIP_2_ Gating in Kir Channels Kir channels were expressed in *Xenopus* oocytes and currents measured in excised inside-out patches. (A) Application of neomycin (Neo) to Kir1.1 channels as indicated; red line represents a monoexponential fit to the time course of recovery from neomycin inhibition; tau_Neo_ value represents mean ± SEM, n = 5. (B) Channel rundown was induced by 1 mM Mg^2+^ and the omission of pyrophosphate and channels were reactivated with diC8-PIP_2_; red line represents a monoexponential fit to the time course of diC8-PIP_2_ reactivation; tau_PIP2_ value represents mean ± SEM, n = 11. (C) Time course of channel reactivation subsequent to complete rundown induced by 3 μM diC8-PIP_2_ (tau_PIP2_ = 16 ± 2 s, n = 5) and 30 μM diC8-PIP_2_ (tau_PIP2_ = 15 ± 3, n = 5); the rates were obtained in the same patch and not significantly different (p > 0.05); red lines represents monoexponential fits to the data. (D) Cartoon indicating that binding of PIP_2_ to the channel is fast and induces a slow conformational change subsequent to PIP_2_ binding that leads to pore opening. The latter process represents the measured diC8-PIP_2_ activation rate.

**Figure 2:**
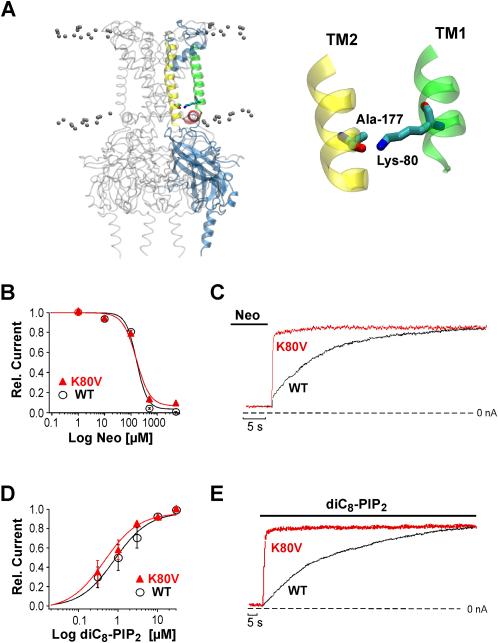
A Residue at the Base of TM1 Controls the Speed of PIP_2_ Activation (A) Side view of the structural model of Kir1.1 (see Experimental Procedures for details) with one of the four subunits highlighted. The highlighted residues correspond to K80 and A177. TM1 is shown in green and TM2 in yellow with the slide helix in red. The expanded right panel shows the proximity and orientation of the side chain of K80 and the backbone carbonyl of A177. (B) Dose-response curves for neomycin inhibition of WT Kir1.1 and K80V channels fitted to a standard Hill function with a IC_50_ = 0.15 ± 0.05 mM and Hill coefficient of 3.7 ± 0.3 (WT) and IC_50_ = 0.16 ± 0.06 mM and Hill coefficient 2.8 ± 0.2 (K80V). Data points represent mean ± SEM of five experiments. (C) Time course of channel reactivation upon removal of 5 mM neomycin for WT and K80V channels. (D) Relative currents (I_diC8-PIP2_/I_before run down_) in response to diC8-PIP_2_ application subsequent to rundown for WT and K80V channels fitted to a standard Hill function with an EC_50_ and Hill coefficient of 0.9 ± 0.1 μM and 0.9 ± 0.1 for WT; 0.7 ± 0.1 μM and 1.1 ± 0.1 for K80V. Data points represent mean ± SEM of seven experiments. (E) Time course of channel reactivation upon application of 3 μM diC8-PIP_2_ for WT and K80V channels.

Although activation by PIP_2_ is common to all eukaryotic Kir channels, very little is known about the kinetics of PIP_2_ activation and the underlying structural mechanism of gating. We therefore measured the time course of channel activation and deactivation in response to a rapid change in membrane PIP_2_ content using two types of protocols. Figure 1A shows the response of Kir1.1 to the application of neomycin, which reversibly complexes membrane PIP_2_. Increasing the neomycin concentration resulted in a fast and stepwise reduction in channel activity with an IC_50_ of 0.15 ± 0.05 mM and complete inhibition at 5 mM (Figures 1A and 2B). Removal of neomycin induced a monoexponential recovery of channel activity with a tau_Neo_ of 18 ± 3 s which represents channel reactivation by the rebinding of PIP_2_ (Figure 1A).

Likewise, subsequent to channel run down (induced by removal of 1 mM pyrophosphate and addition of 1 mM Mg^2+^ to the bath solution to promote breakdown of endogenous PIP_2_) application of short chain PIP_2_ (diC8-PIP_2_) resulted in stepwise channel reactivation with about 80% activation for 3 μM diC8-PIP_2_ and a corresponding EC_50_ of 0.9 ± 0.1 μM (Figures 1B and 2D). Channel reactivation by diC8-PIP_2_ followed a monoexponential time course with a tau_PIP2_ of 27 ± 3 s for 3 μM diC8-PIP_2_ (Figure 1B). Importantly, increasing the diC8-PIP_2_ concentration to 30 μM had no effect on the speed of activation (Figure 1C) indicating that it is not PIP_2_ binding which is rate limiting for channel activation, but instead a subsequent conformational change (see cartoon in Figure 1D). The time course for channel reactivation induced by neomycin removal (tau_Neo_ = 18 ± 3 s) and diC8-PIP_2_ application (tau_PIP2_ = 27 ± 3 s) were similar suggesting that the activation process we observe is similar for both endogenous PIP_2_ and exogenous diC8-PIP_2_.

### A Residue in TM1 Controls the Speed of PIP_2_ Activation

Figure 2A shows the position of lysine 80 in TM1 at the helix bundle crossing in a structural model of the Kir1.1 channel. This residue controls the ability of Kir1.1 channels to close in response to intracellular acidosis by an unknown allosteric mechanism. We therefore examined the role of K80 in PIP_2_ gating. Mutation of K80 to valine (K80V) had little effect on the PIP_2_ affinity as the concentration dependence for neomycin inhibition and diC8-PIP_2_ activation were similar for K80V and wild-type (WT) channels (Figures 2B and 2D). However, K80V dramatically altered the kinetics of PIP_2_ activation. The speed for activation induced by 3 μM diC8-PIP_2_ (tau_PIP2_ = 0.6 ± 1 s) or neomycin removal (tau_Neo_ = 0.3 ± 0.1 s) was more than 45-fold faster compared to WT channels (Figures 2C and 2E). As observed in WT channels the rate of PIP_2_ activation in K80V channels was similar for 3 μM and 30 μM diC8-PIP_2_ (data not shown). The rate for channel deactivation upon diC8-PIP_2_ removal in WT Kir1.1 channels was more variable and was faster (between 2 s and 21 s, see Figure 1C) than the PIP_2_ activation rate but was not significantly different for WT and K80V channels (p > 0.05). The variability in the deactivation time course most likely reflects differences in the washout time course of diC8-PIP_2_ from the patch and, thus, likely not the deactivation gating kinetics of the channel.

This observation that the K80V mutation dramatically altered the kinetics of PIP_2_ activation without affecting PIP_2_ affinity strongly supports the idea that the time course of PIP_2_ activation represents a relatively slow conformational change that takes place subsequent to the PIP_2_ binding step (as depicted in Figure 1D).

### H Bonding at the Helix-Bundle Crossing Determines PIP_2_ Activation Kinetics in Kir1.1

**Figure 3:**
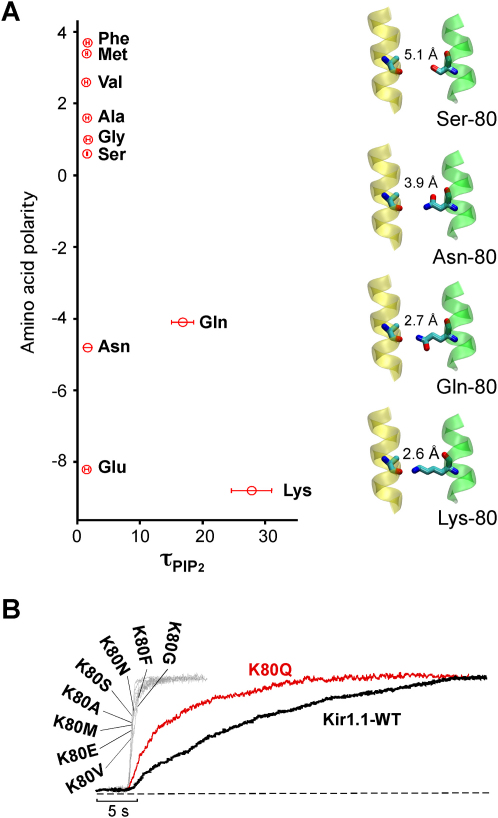
TM1-TM2 H Bonding Determines the Rate of PIP_2_ Activation (A) Amino acid polarity (Engelman et al., 1986) plotted against the tau of channel activation (mean ± SEM of at least eight experiments) obtained with 3 μM diC8-PIP_2_ subsequent to complete rundown of Kir1.1 channels and the indicated mutants at the K80 position; note that there is no correlation between polarity and PIP_2_ activation rate. The right-hand panels depict the side chain of A177 with K80, K80Q, K80N, and K80S as well as the calculated minimum distance between the carbonyl oxygen of A177 and the terminal heavy atom of the residue at position 80. TM1 is highlighted in green and TM2 in yellow. (B) Time course of channel activation by 3 μM diC8-PIP_2_ for Kir1.1 WT and mutant channels as indicated.

We examined the effect of various residues at position 80 on the rate of PIP_2_ activation. Mutation of K80 to valine (K80V), methionine (K80M), alanine (K80A), phenylalanine (K80F), serine (K80S), glycine (K80G), asparagine (K80N), and glutamate (K80E) all resulted in fast diC8-PIP_2_ activation with tau_PIP2_ values ranging from 0.6 to 0.8 s. The K80R mutation was nonfunctional (data not shown). These results indicate that neither the size nor polarity of the residue correlated with the speed of PIP_2_ activation (Figures 3A and 3B). Furthermore, no obvious change in the deactivation time course upon diC8-PIP_2_ removal was observed (data not shown) suggesting that these mutations primarily affected the activation process. However, mutation of lysine 80 to a glutamine (K80Q) resulted in a slow rate of activation with a corresponding tau_PIP2_ of 16 ± 2 s. This suggested that the −NH_2_ group present in both the lysine and glutamine side chains might be a structural requirement for the slow-gating phenotype.

To better understand these findings from a structural perspective, we employed a combination of homology modeling and molecular dynamics simulations. We used a homology model of Kir1.1 created previously which was based upon the recently determined structures of KirBac1.1, KirBac3.1, and the intracellular domains of the Kir2.1 and Kir3.1 channels (Rapedius et al., 2006).

Figure 2A depicts the location and orientation of Lys-80 and shows that the side chain ɛ-nitrogen points directly at the backbone carbonyl oxygen of alanine 177 in TM2 of the same subunit. The distance between these atoms varied from 2.8 to 3.0 Å in the four subunits in our closed-state model. This proximity is compatible with the formation of a hydrogen (H) bond between Lys-80 and Ala-177 (hereafter referred to as the TM1-TM2 H bonding).

In principle, serine (Ser-80), asparagine (Asn-80), and glutamine (Gln-80) could also support a TM1-TM2 H bridge at this position. We therefore substituted different residues into position 80 of our homology model and examined their ability to form hydrogen bonds over a range of distances during a molecular dynamics simulation (see Experimental Procedures and see Figure S1 in the Supplemental Data available with this article online). The simulations revealed that the lysine ɛ-nitrogen approached as close as 2.6 Å to the carbonyl oxygen of Ala-177, and was readily able to form H bonds, spending approximately 50% of the time H bonded at 310 K. By contrast, the asparagine nitrogen and serine oxygen only approached as close as 3.9 Å and 5.1 Å, respectively, owing to their shorter side chain length (depicted in Figure 3). Therefore neither residue is capable of forming H bonds as the length of a H bond is typically ≤3.0 Å. However, glutamine could approach as close as 2.7 Å but less frequently than lysine and, correspondingly, spent 5% of the time H bonded (see Figure S1).

Even though these simulations are an oversimplification of the in vivo situation, they nevertheless suggest that only Lys-80 and Gln-80 are capable of supporting TM1-TM2 H bonding in the closed-state model, which correlates qualitatively with the slow rates of PIP_2_ activation seen for Gln-80 (tau_PIP2_ = 16 ± 2 s) and Lys-80 (tau_PIP2_ = 27 ± 3 s). Correspondingly, all those residues with fast rates of PIP_2_ activation are either incapable of supporting an H bond because there are no hydrogen donors (Met, Ala, Val, Phe, Gly, Glu) or do not approach close enough (Asn and Ser).

This apparent correlation between TM1-TM2 H bonding ability and the PIP_2_ activation rate suggests that the activation energy for channel opening is determined by the ability of TM1-TM2 H bonding in the closed state. This is supported by a recent model of the open state of KirBac3.1, which demonstrated a large movement between TM1 and TM2 at the helix bundle crossing which would be expected to rupture any TM1-TM2 H bonding upon channel opening (see Discussion).

### A Conserved Gating Motif at the Helix Bundle Crossing

**Figure 4:**
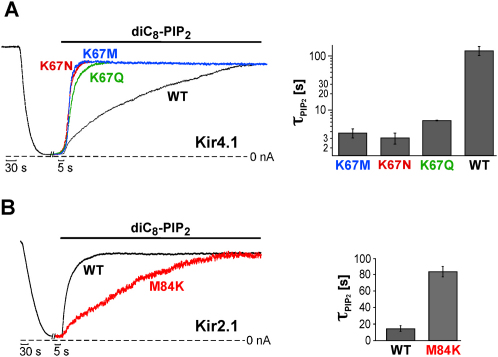
Conserved Role of TM1-TM2 H Bonding for Kir Channel PIP_2_ Gating (A) Time course for Kir4.1 channel rundown (induced by poly-lysine, only WT shown) and reactivation by 30 μM diC8-PIP_2_ for WT and indicated mutants. Right panel: bars represent tau_PIP2_ (mean ± SEM of at least five experiments) for diC8-PIP_2_ activation (30 μM) for WT and Kir4.1 mutants. (B) Time course for Kir2.1 channel rundown induced by 1 mM Mg^2+^ and the omission of pyrophosphate (only WT shown) and channel reactivation with 3 μM diC8-PIP_2_ for WT and with 30 μM diC8-PIP_2_ for M84K. Right panel: bars represent tau_PIP2_ (mean ± SEM of at least five experiments) for diC8-PIP_2_ activation (3 μM) for WT and M84K (30 μM).

Kir4.1 also has a lysine (Lys-67) at the homologous position to K80 in Kir1.1. We therefore investigated whether Kir4.1 demonstrates a similar mechanism of PIP_2_ gating. Figure 4A shows that WT Kir4.1 also displays very slow diC8-PIP_2_ activation (tau_PIP2_ = 123 ± 23 s) consistent with a TM1-TM2 H bridge (Figure 4A). Mutation of this lysine to methionine (K67M), to asparagine (K67N) and to glutamine (K67Q) dramatically sped up diC8-PIP_2_ activation with corresponding tau_PIP2_ of 3.7 ± 0.7 s, 3 ± 0.7 s, and 6.3 ± 0.1 s, respectively (Figure 4A). Thus, in Kir4.1, only lysine appeared to show significant TM1-TM2 H bonding, although the somewhat slower rate of K67Q compared to K67M/N might indicate some H bonding. Thus the equivalent mutations in Kir4.1 also support a model in which TM1-TM2 H bonding at the helix-bundle crossing controls the rate of PIP_2_ activation.

This idea is also supported by the observation that wild-type Kir2.1, which possesses a methionine at this position (Met-84), was activated by diC8-PIP_2_ with a tau_PIP2_ of 14 ± 3 s, whereas M84K channels had a markedly slower rate of PIP_2_ activation (tau_PIP2_ = 84 ± 6 s). This result is consistent with the introduction of a TM1-TM2 H bridge in Kir2.1-M84K and suggests that the mechanism of gating at the helix-bundle crossing is highly conserved in the Kir channel superfamily.

### TM1-TM2 H Bonding Also Controls pH Sensitivity

**Figure 5:**
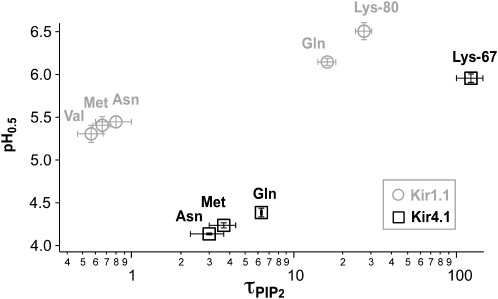
TM1-TM2 H Bonding Determines the pH Sensitivity of Kir Channels IC_50_ for pH inhibition (pH_0.5_) plotted for Kir1.1 WT (Lys-80) and indicated mutants and also Kir4.1 WT (Lys-67) and indicated mutants against the respective tau_PIP2_ for diC8-PIP_2_ activation as determined in Figures 3 and 4. The IC_50_ values for the Kir4.1 mutants represent an estimation because the low pH sensitivity of these mutants meant a complete dose-response curve could not be obtained (see Figure S2).

The presence of a lysine residue at the TM1 site has also been shown to determine the differences in Kir channel pH sensitivity. We therefore examined whether the proposed TM1-TM2 H bonding represents the structural basis for this by measuring the pH sensitivity of different K80 mutations in Kir1.1 and K67 mutations in Kir4.1 (Figure S2) and plotted these values against the tau_PIP2_ for PIP_2_ activation (Figure 5). This plot reveals a clear correlation between the pH sensitivity (IC_50_ values) and the PIP_2_ activation rate (tau_PIP2_). In Kir1.1, the mutations K80V, K80M, and K80N all show low pH sensitivity (IC_50_ ≈5.3). By contrast, K80Q had an intermediate pH sensitivity (IC_50_ = 6.1 ± 0.1) and WT Kir1.1 the highest pH sensitivity (IC_50_ = 6.5 ± 0.1). This correlates well with the rates of PIP_2_ activation (K80M = K80N >> K80Q > K80) and, thus, the TM1-TM2 H-bonding ability.

A similar correlation was also observed for the pH sensitivity of Kir4.1 with low pH sensitivities for K67M, K67N, and K67Q with estimated IC_50_ values around 4.2 (Figure S2) and high pH sensitivity for WT (5.95 ± 0.05). Again, these values correlated with the PIP_2_ activation rates that were markedly faster for K67N, K67M, and K67Q compared to WT. Furthermore, WT-Kir2.1 exhibited a low pH sensitivity (IC_50_ = 5.3 ± 0.1) and relatively fast PIP_2_ activation (tau_PIP2_ = 14 ± 3 s), whereas Kir2.1-M84K displays a high pH sensitivity (IC_50_ = 7.0 ± 0.2) and a markedly slower rate of PIP_2_ activation (tau_PIP2_ = 84 ± 6 s). These results suggest that this TM1-TM2 H bonding is the primary structural determinant for Kir channel pH sensitivity.

A straightforward explanation is that protonation in the cytoplasmic domains induces channel closure and that H-bonding at the helix-bundle crossing stabilizes this closed state. If this hypothesis is correct, then this should also be reflected in the kinetics of pH gating and predicts a slowing in the rate of recovery from pH inhibition in Kir channels which possess TM1-TM2 H bonding at the helix-bundle crossing.

### Control of pH Gating Kinetics by H Bonding at the Helix-Bundle Crossing

**Figure 6:**
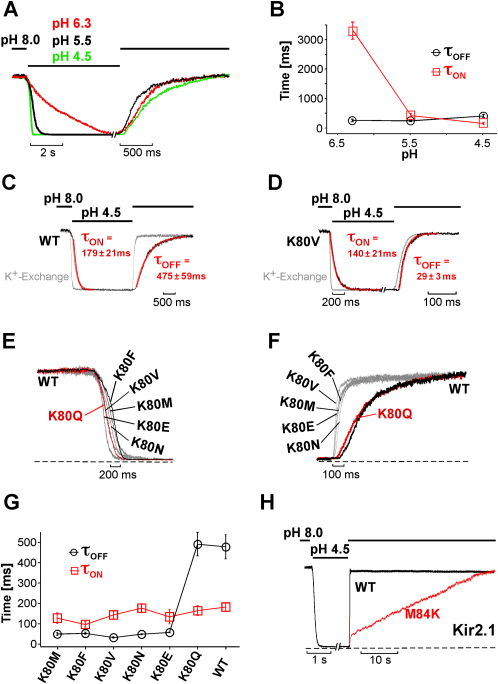
TM1-TM2 H Bonding Controls pH Gating in Kir Channels (A) Time course of pH gating in Kir1.1 channels induced by changes in the intracellular pH established by a fast piezo-driven application system. Currents for the different pH values were equalized for better comparison (i.e., at pH 6.3 only 60% of the current was inhibited). (B) The inhibition and recovery time course from experiments as shown in (A) were fitted with monoexponential functions and tau values for pH inhibition (τ_on_) and recovery from pH inhibition upon alkalization pH 8.0 (τ_off_) are plotted. Data points represent mean ± SEM of at least six experiments. (C) Time course of Kir1.1 currents upon K^+^ exchange (replacement with Na^+^ measured at +40 mV) are shown in gray and superimposed on the pH gating time course obtained in the same patch; red lines represent monoexponential fits for pH inhibition (τ_on_) (mean ± SEM, n = 6) and recovery (τ_off_) (mean ± SEM, n = 6). (D) Time course of Kir1.1-K80V currents upon K^+^ exchange (replacement with Na^+^ measured at +40 mV) are shown in gray and superimposed on the pH gating time course obtained in the same patch; red lines represent monoexponential fits for pH inhibition (τ_on_) (mean ± SEM, n = 7) and recovery (τ_off_) (mean ± SEM, n = 7). (E) Time course of pH inhibition (pH 4.5) for Kir1.1-K80 mutants and WT. (F) Time course of recovery from pH inhibition (pH 4.5) upon alkalization (pH 8.0) for Kir1.1-K80 mutants and WT. (G) From experiments such as in (E) and (F), the tau values for pH inhibition at pH 4.5 (τ_on_) and recovery at pH 8.0 (τ_off_) are plotted. Data points represent mean ± SEM of at least five experiments. (H) Time course of pH inhibition and recovery for Kir2.1 and Kir2.1-M84K channels.

Figure 6A shows the time course of H^+^ inhibition in Kir1.1 channels for different pH jumps and channel recovery upon alkalization to pH 8.0 using a piezo-driven fast solution exchange system. The inhibition and recovery kinetics displayed monoexponential behavior. The tau value for H^+^ inhibition increased from 3366 ± 528 ms, to 455 ± 25 ms and 179 ± 21 ms for pH jumps to 6.3, 5.5, and 4.5, respectively, corresponding to 61% ± 3%, 92% ± 1% and 93% ± 1% of inhibition (Figures 6A and 6B). By contrast, channel recovery upon alkalization was independent of the pH used to induce inhibition (Figures 6A and 6B). In the same patches, the speed of solution exchange as determined with a K^+^/Na^+^ replacement protocol was found to be much faster than the measured pH gating kinetics (tau ≈30 ms; Figure 6C). Thus, assuming that channel protonation/deprotonation is very rapid, the measured channel gating kinetics should reflect the conformational change subsequent to the protonation/deprotonation steps.

Intriguingly, we found that the recovery rates from pH inhibition were markedly faster in mutants with K80 replaced by M, F, V, N, and E with corresponding tau values between 20 and 60 ms (Figures 6F and 6G). In these fast-gating mutants, recovery from pH inhibition closely followed the time course of the solution exchange (Figure 6D), and thus, the real recovery-gating rates might actually be considerably faster. Consistent with our hypothesis, substitution of K80 by glutamine (K80Q) resulted in a slow recovery similar to WT (Figures 6F and 6G). Furthermore, the speed of H^+^ inhibition was similar for WT and various K80 mutants (Figures 6E and 6G), suggesting that these mutations primarily affected the closed to open transition.

Consistent with these observations, Kir2.1 recovery from pH inhibition was fast (tau = 36 ± 5 ms) but dramatically slowed upon introduction of the TM1 lysine (M84K; Figure 6H). The recovery time course in M84K channels appeared to be more linear than exponential and within 18 ± 1 s half of the channel activity recovered. In some patches a small (<20%) faster initial phase was seen similar to that shown in Figure 6H. However, this fast phase was still more than 10-fold slower (tau = 385 ± 21 ms) than recovery in WT channels.

As expected, recovery from pH inhibition in Kir4.1 channels was slow (tau = 4321 ± 566 ms). However, for the mutants K67M, K67N, and K67Q, a meaningful determination of the pH gating kinetics was not possible because pH inhibition was very weak (around 20% at pH 4.5; Figure S2) and an initial activation occurred prior to the onset of inhibition (data not shown).

In summary, recovery from pH inhibition was slow in Kir channels with TM1-TM2 H bonding (e.g., Kir1.1-WT/K80Q, Kir2.1-M84K) and fast in mutants lacking H bonding (e.g., Kir1.1-K80V, Kir2.1 WT), strongly supporting the concept of a conserved gating mechanism at the helix-bundle crossing for pH and PIP_2_ gating in Kir channels that is controlled by TM1-TM2 H bonding.

### Gating at the Selectivity Filter

pH inhibition in Kir1.1 is tightly coupled to a K^+^-dependent conformational change at the extracellular pore, i.e., in the absence of extracellular permeant ions (e.g., K^+^, Rb^+^, Cs^+^), the channels enter a nonconductive state subsequent to pH inhibition which is due to a collapse of the pore at the selectivity filter (Dahlmann et al., 2004; Schulte et al., 2001). We therefore investigated whether this K^+^-dependent inactivation process can also be initiated by PIP_2_ depletion and whether TM1-TM2 H bonding is involved.

**Figure 7:**
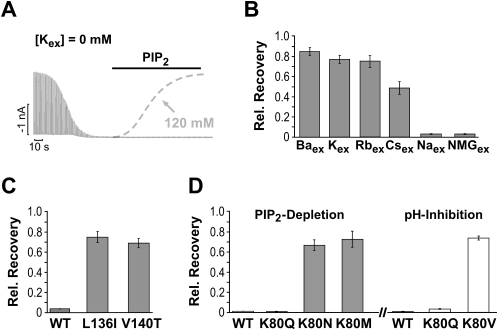
PIP_2_ Depletion Induces K^+^-Dependent Inactivation Process at the Selectivity Filter (A) Rundown of Kir1.1 channels and lack of reactivation by 25 μM PIP_2_ in the absence of extracellular K^+^ (120 mM NMG^+^ solution), gray dotted line represents the time course of PIP_2_ reactivation typically obtained with 120 mM extracellular K^+^. (B) Bars represent normalized channel recovery induced by heparin (removal of poly-lysine, see Figure S3) subsequent to 45 s of channel inhibition by poly-lysine with extracellular 10 mM Ba^2+^ (added to a NMG^+^ solution), 120 mM K^+^, 120 mM Rb^+^, 120 mM Cs^+^, 120 mM Na^+^, and 120 mM NMG^+^. Data points represent mean ± SEM of at least four experiments. (C) Bars represent normalized channel recovery induced by heparin subsequent to 45 s of channel inhibition by poly-lysine with extracellular 120 mM NMG^+^ (zero K^+^ solution) for Kir1.1 WT, L136I, and V140T channels. Data points represent mean ± SEM of at least five experiments. (D) Dark bars represent normalized channel recovery induced by heparin (removal of poly-lysine) subsequent to 45 s of channel inhibition by poly-lysine with extracellular 120 mM NMG (zero K^+^ solution) for Kir1.1 WT, K80Q, K80N, and K80M channels; white bars represent normalized channel recovery induced by pH 8.0 subsequent to 45 s of H^+^ inhibition with extracellular 120 mM NMG^+^ (zero K^+^ solution) for Kir1.1 WT (pH 6.0), K80Q (pH 5.5), and K80V (pH 4.5). Data points represent mean ± SEM of at least four experiments.

Figure 7A shows that PIP_2_ depletion (normal run down) resulted in a permanent loss of Kir1.1 activity in the absence of extracellular permeant ions, i.e., addition of PIP_2_ could not recover channel activity. The same results were also obtained using poly-lysine to induce PIP_2_ depletion (Figure S3). By contrast, in the presence of high extracellular permeant ions (e.g., K^+^, Rb^+,^ and Cs^+^) activity was readily restored by PIP_2_ (Figure 7B). Likewise, Ba^2+^, which also binds in the selectivity filter, abolished K^+^-dependent inactivation (Figure 7B). Furthermore, the rate of K^+^-dependent channel inactivation induced by PIP_2_ depletion (tau = 15 ± 1 s) was similar to that obtained by pH inhibition (tau = 7 ± 1 s; Figure S3), suggesting that PIP_2_ depletion and pH inhibition both induce a similar inactivation process. This concept was further strengthened by the finding that mutations within the P loop (L136I, V140T) that abolish pH induced K^+^-dependent inactivation (Schulte et al., 2001) also abolished inactivation induced by PIP_2_ depletion (Figure 7C).

We next investigated whether TM1-TM2 H bonding is required for this K^+^-dependent inactivation process in Kir1.1. Figure 7D shows that both WT and K80Q channels (showing H bonding), but not K80M or K80N channels (lacking H bonding), exhibited K^+^-dependent inactivation in response to PIP_2_ depletion. Likewise, K80Q channels also exhibited pH-induced K^+^-dependent inactivation, but not K80V.

These results demonstrate that the proposed TM1-TM2 H bonding at the helix-bundle crossing is essential for mediating the conformational changes within the selectivity filter which are thought to represent a collapse of the selectivity filter.

## Discussion

### Dissecting the Kinetics of PIP_2_ Gating in Kir Channels

In this study, we have established an assay which can measure the kinetics of Kir channel gating produced by a rapid change in the membrane PIP_2_ concentration. Two protocols were used: (1) fast application of exogenous diC8-PIP_2_ subsequent to depletion of endogenous PIP_2_ (run down) and (2) sequestration of endogenous membrane PIP_2_ with neomycin followed by its rapid removal which results in a sudden increase in endogenous membrane-bound PIP_2_. Both protocols resulted in monoexponential reactivation of Kir1.1 channel activity with similar rates (tau_PIP2_ = 27 ± 3 s; tau_Neo_ = 18 ± 3 s). Two lines of evidence indicate that PIP_2_ binding to the channels is fast and that the measured activation rate is determined by a slow and rate-limiting conformational change which occurs subsequent to PIP_2_ binding. First, the PIP_2_ activation kinetics are not affected by the concentration of diC8-PIP_2_. Second, mutations in Kir1.1 (e.g., K80V) dramatically increased the rate of channel activation (tau_PIP2_ = 0.6 ± 1 s; tau_Neo_ = 0.3 ± 0.1 s) without affecting PIP_2_ affinity, strongly suggesting that these mutations affect a conformational step subsequent to the PIP_2_-binding step. In other words, in response to a sudden jump in the membrane PIP_2_ content, PIP_2_ binds rapidly to the channel (faster than 0.3 s) and then induces a slow conformational change which represents the measured activation rate (as depicted in Figure 1D).

For WT Kir1.1, deactivation upon diC8-PIP_2_ removal or neomycin application was considerably faster (about 5-fold) than the rate of activation (Figure 1C). Thus, the rates of activation and deactivation cannot be explained by a simple bimolecular reaction scheme, as this would predict a faster opening rate than closing rate given that activation was almost complete. Further, mutations (e.g., Kir1.1-K80V) dramatically increase the speed of PIP_2_ activation without affecting the closed- to open-state equilibrium for a given PIP_2_ concentration. This suggests a more complicated reaction scheme with several closed states where transitions that control the kinetics of PIP_2_ activation have little impact on the overall open probability. A similar situation has been reported for Kv channels where mutations that dramatically speed up the activation kinetics have little effect on the steady-state voltage dependence (Scholle et al., 2004). The existence of several closed states in the PIP_2_ gating pathway also seems likely given the tetrameric structure of the Kir channel and four possible PIP_2_-binding sites.

The ability to dissect the kinetics of PIP_2_ gating should now allow a detailed insight into the PIP_2_ gating mechanism by addressing important questions such as PIP_2_ binding stoichiometry and subunit cooperativity, e.g., by using channels with fixed stoichiometries of slow- (e.g., WT) and fast-gating (e.g., K80M) subunits.

### Comparison of the pH and PIP_2_ Gating Kinetics

Here, we also present the first measurements of the kinetics of pH gating by utilizing a fast application system. For WT Kir1.1, the tau for H^+^ inhibition increases with higher H^+^ concentrations from 3366 ± 528 ms at pH 6.3, to 455 ± 25 ms at pH 5.5, and to 179 ± 21 ms at pH 4.5. By contrast, recovery from H^+^ inhibition upon alkalization (pH 8.0) was unaffected by pH. Given that channel protonation should be very rapid and that the solution exchange was sufficiently faster than the channel-gating kinetics, we can assume that the measured rates reflect the channel gating kinetics subsequent to the protonation and deprotonation steps.

Similar to the effects seen during PIP_2_ gating, the steady-state situation does not simply relate to the on and off rates since at pH 6.3 inhibition is much slower than recovery even though about 60% inhibition is obtained. This, again, points to a more complicated gating mechanism that awaits further detailed characterization. Yet despite these similarities, there are still important differences between the pH and PIP_2_ gating mechanisms. First, the K80V mutation speeds the reopening kinetics and markedly reduces the pH sensitivity but does not alter the dose-response curve for PIP_2_ activation. Another difference is the rate of channel reopening itself, which is much faster for pH gating compared to PIP_2_ gating, indicating that the conformational changes (probably in the cytoplasmic domains) induced by either deprotonation or PIP_2_ binding occur at different speeds. However, at the helix-bundle crossing the conformational changes appear similar since the kinetics of pH gating and PIP_2_ gating are both controlled by the residue at the TM1 site, i.e., by TM1-TM2 H-bonding ability (see below).

The ability to now measure the kinetics of PIP_2_ gating represents an opportunity to separate effects on PIP_2_ *binding* versus effects on PIP_2_ *efficacy*, i.e., whether a mutation affects PIP_2_ gating by directly affecting binding, or by indirectly affecting the steps subsequent to PIP_2_ binding, or both (Colquhoun, 1998). Likewise, knowledge of the pH gating kinetics might help to gain an insight into the mechanisms that underlie the changes in pH sensitivity reported for many Kir channel mutants. Therefore, the ability to study both the pH and PIP_2_ gating kinetics will provide a better understanding of mutations that produce Kir channelopathies by affecting the regulation by PIP_2_ and pH (Donaldson et al., 2003; Lin et al., 2006; Lopes et al., 2002; Ma et al., 2006). Furthermore, we demonstrate that neomycin and diC8-PIP_2_ are useful tools to induce very rapid changes in the membrane PIP_2_ content. This could be highly valuable to the study of other classes of ion channels that are also regulated by PIP_2_ (Hilgemann et al., 2001; Nilius et al., 2007, Suh et al., 2006; Zolles et al., 2006).

### Control of PIP_2_ and pH Gating by a Putative TM1-TM2 H Bond

**Figure 8:**
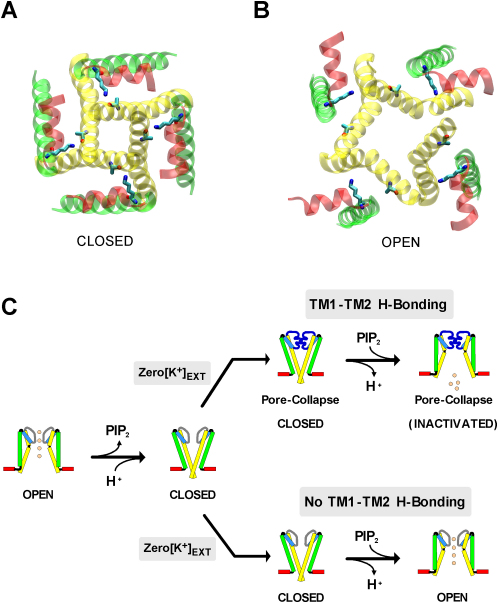
Gating Models of Kir1.1 (A) Bottom-up view through the pore of the closed-state structure of Kir1.1. TM1 is shown in green, TM2 in yellow, and the slide helix in red. Residues Lys-80 and Ala-177 are shown as sticks and colored CPK. The H bond between the ɛ-nitrogen of Lys-80 and the backbone carbonyl oxygen of Ala-177 is thought to stabilize the closed state of the channel. (B) The predicted open-state structure of the pore of Kir1.1. This model is based upon the open-state structure of KirBac3.1 and highlights the relative movement between the TM helices. The minimum distance between Lys-80 and Ala-177 increases from <3 Å to >12 Å in the open state and would therefore rupture any H bond between these residues upon channel opening. The relative movement between the helices can be better visualized in the accompanying movie (see Supplemental Data, Movie S1). (C) Cartoon depicting the contribution of the TM1-TM2 H bond to collapse of the pore. TM1 is shown in green, TM2 in yellow, and the slide helix in red. Either PIP_2_ depletion or channel protonation induces channel closure at the helix-bundle crossing. With extracellular K^+^ removed (zero [K^+^]_ext._), closed channels enter an inactivated state (pore collapse at the selectivity filter) in channels with TM1-TM2 H bonding (e.g., WT Kir1.1 or K80Q). In this situation the channel remains in an “inactivated state,” even when PIP_2_ is reapplied or the channel is deprotonated (upper gating pathway). However, in channels with no TM1-TM2 H bonding (e.g., K80V), inactivation is not possible and channels can open again upon the rebinding of PIP_2_ or channel deprotonation even in the absence of extracellular K^+^ (lower gating pathway). Note: the gate at the helix bundle crossing in the inactivated state (upper gating pathway) is shown to be open; however, this is just an assumption and requires further investigation.

Although an atomic resolution structure of Kir1.1 is ultimately required to prove this hypothesis, our collective findings provide compelling evidence that a hydrogen bond may exist between the ɛ-nitrogen of Lys-80 in Kir1.1 and the backbone carbonyl oxygen of Ala-177 at the helix bundle crossing and that this TM1-TM2 H bond represents an important structural determinant of Kir channel gating by PIP_2_ and pH. The key observation was the striking correlation between the ability for TM1-TM2 H bonding in several different Kir channels (Kir1.1, Kir4.1, and Kir2.1) and their rate of channel opening induced by changes in either PIP_2_ or pH. The fact that the TM1-TM2 H bond affected the rate of channel opening, but not closing, suggests that rupture of this H bond occurs upon channel opening. In agreement with this, recent structures of KirBac3.1 in both the closed state and the open state indicate a large rotation and tilt of the TM1 and TM2 helices during channel opening (Kuo et al., 2005, Domene et al., 2005). We therefore used this open-state model of KirBac3.1 to produce a homology model of the Kir1.1 TM-helices in the open state (Figure 8B).

This model shows that the distance between the ɛ-nitrogen of K80 and backbone oxygen of A177 increases to >12 Å in the open state and that such movement would clearly rupture any H bond between K80 and A177 as the channel moved into the open state (Figures 8A and 8B). These TM1-TM2 movements are better visualized in the accompanying movie which depicts the hypothetical transition between closed- and open-state models (see Supplemental Data, Movie S1).

Our observation that the effects of the proposed TM1-TM2 H bonding are conserved in both Kir1.1 and Kir4.1, and can also be transferred to Kir2.1 demonstrates a high degree of functional and structural conservation of the gating machinery in the Kir channel superfamily. The study also represents an important functional validation of the current models of Kir channel structure, including the proposed open-state structure of the KirBac channel.

### The Structural Determinant of Kir Channel pH Sensitivity

It is well known that the presence of a lysine in TM1 (e.g., Kir1.1-K80) is a major determinant of pH sensitivity in all Kir channels (Schulte and Fakler, 2000). However, the precise mechanism underlying the importance of this residue has remained elusive (Rapedius et al., 2006). This study now presents a straightforward mechanistic answer; residues at this position (Lys and Gln) that can support H bonding produce channels with high pH sensitivity, whereas residues which cannot H bond produce channels with low pH sensitivity.

We therefore propose a model in which protonation of the intracellular domains induces conformational changes that are transduced into gate closure at the helix-bundle crossing and that the pH-induced closed state is stabilized by TM1-TM2 H bonding. In strong support of this, the time course of recovery from pH inhibition was slow in Kir channels with TM1-TM2 H bonding (e.g., Kir1.1 WT and Kir2.1-M84K) but fast in Kir channels lacking TM1-TM2 H bonding (e.g., Kir1.1-K80M and Kir2.1), consistent with the concept that rupture of this H bond contributes to the activation energy for the closed- to open-state transition.

### H Bonding at the Helix-Bundle Crossing Gates the Selectivity Filter

PIP_2_ depletion triggers an inactivation process in Kir1.1 channels that depends on the extracellular ion composition. In the absence of extracellular ions that can enter the selectivity filter (e.g., K^+^, Rb^+^, Cs^+^, and Ba^2+^), PIP_2_ depletion lead to irreversible pore closure, i.e., rebinding of PIP_2_ could not recover channel activity. A similar ionic sensitivity has been reported when Kir1.1 is inhibited by low intracellular pH (Dahlmann et al., 2004; Schulte et al., 2001). Furthermore, mutations in the P loop which abolish this pH-induced K^+^-dependent inactivation (Schulte et al., 2001) also abolished PIP_2_-depletion-induced inactivation, demonstrating a common structural basis for this K^+^-dependent inactivation process.

Intriguingly, we observed that K^+^-dependent inactivation in Kir1.1 also depends on the H bonding at the helix-bundle crossing. This demonstrates that interactions at this site are able to influence the selectivity filter. In other words, the TM1-TM2 H bond places the TM helices into a conformation that promotes the collapse of the selectivity filter (as depicted in Figure 8C). The fact that this inactivation only proceeds from the closed, not the open state, is also consistent with our model in which this H bond cannot form in the open state.

It will be interesting to establish how closely this mechanism of long range coupling between the helix-bundle crossing and the selectivity filter relates to the K^+^-dependent C-type inactivation seen in many Kv channels, HERG channels, and KcsA, and which is also thought to involve a structural change in the selectivity filter induced by a movement in the TM helixes (Baukrowitz and Yellen, 1996; Cordero-Morales et al., 2006; Loots and Isacoff, 2000; Smith et al., 1996, Starkus et al., 1997; Yellen, 1998).

Physiologically, this extracellular K^+^ dependence of Kir1.1 channels represents a renal feedback system to prevent excessive K^+^ loss during hypokalaemia (Dahlmann et al., 2004; Schulte et al., 2001). Here, we report that PIP_2_ depletion also sensitizes Kir channels to changes in the extracellular K^+^. This could be of significant physiological relevance since dynamic Kir1.1 regulation by PIP_2_-related mechanisms has also been observed in renal epithelia (Zeng et al., 2003) and could therefore also be important for the functional role of neurons in chemosensitive areas of the brainstem which express Kir1.1 (Wu et al., 2004).

### Conclusions

We report that PIP_2_ binding induces a conformational change in Kir channels which initiates at the cytoplasmic domains, followed by a movement of the TM helices that is controlled by a putative H bond at the helix-bundle crossing. This proposed structural mechanism also appears to be highly conserved during both pH gating and PIP_2_ gating in several different Kir channels, thus pointing to an evolutionary conserved gating machinery in the Kir superfamily.

We have also established that this Kir channel gating motif determines their pH sensitivity, their kinetics of PIP_2_ and pH gating, and also links movements at the helix-bundle crossing to the structural changes within the selectivity filter. Many questions still remain elusive such as subunit cooperativity, the stoichiometry of PIP_2_ binding, and the detailed gating schemes for PIP_2_ and pH gating. However, the approaches described here for the direct measurement of Kir channel gating kinetics will now allow these questions to be addressed in the near future.

## Experimental Procedures

### Mutagenesis, cRNA Synthesis, and Oocytes Injection

Rat Kir1.1, rat Kir2.1, and rat Kir4.1 were used in this study. Site directed mutagenesis was performed using the QuikChangeII system (Stratagene) and verified by sequencing. For oocyte expression, constructs were subcloned into the pBF expression vector. mRNAs were synthesized in vitro by using the SP6 mMESSAGE mMACHINE kit (Ambion) and stored in stock solutions at −80°C. *Xenopus* oocytes were surgically removed from adult females and manually dissected. About 50 nl of a solution containing channel specific mRNA was injected into Dumont stage VI oocytes. Oocytes were treated with 0.5 mg/ml collagenase type II (Sigma), defolliculated, and incubated at 19°C for 1–3 days prior to use.

### Electrophysiology

Giant patch recordings in inside-out configuration under voltage-clamp conditions were made at room temperature 3–7 days after mRNA injection. Pipettes were made from thick-walled borosilicate glass, had resistances of 0.3–0.9 MΩ (tip diameter of 5–15 μm), and filled with (in mM, pH adjusted to 7.2 with KOH) 120 KCl, 10 HEPES, and 1.8 CaCl_2_. K^+^-free solution contained (pH adjusted to 7.2 with NaOH) 120 mM N-methylglucamine (NMG^+^), 10 mM HEPES, and 1.8 mM CaCl_2_. Currents were recorded with an EPC9 amplifier (HEKA electronics, Lamprecht) and sampled at 1 kHz with analog filter set to 3 kHz (−3 dB). Solutions were applied to the cytoplasmic side of excised patches via a multibarrel pipette (experiments on PIP_2_ gating and K^+^-dependent inactivation). For experiments on the pH gating kinetics a faster solution exchange system was used with double barrel theta-glass capillary attached to a piezo-driven device. The intracellular solution had the following composition (K_int_): 120 mM KCl, 10 mM HEPES, 2 mM K_2_EGTA, and 1 mM Na pyrophosphate, adjusted to the appropriate pH with HCl. Neomycin, poly-lysine, heparin, and long-chain L-α-Phosphatidyl-D-myo-inositol-4,5-phosphate (PIP_2_) were purchased from Sigma-Aldrich. Short-chain (diC8) L-α-Phosphatidyl-D-myo-inositol-4,5-phosphate was purchased from AG Scientific. All substances were stored as stocks (1–10 mM) at −80°C and diluted in K_int_ solution to final concentrations; long-chain PIP_2_ was sonicated for 15 min and used within 2–6 hr.

### Assessing the Degree of Interaction between K80 and A177 in the Closed State of Kir1.1

The coordinates for Ala-177 and Lys-80 were removed from the Kir1.1 closed-state model (Rapedius et al., 2006) and, where necessary, the lysine was mutated to another amino acid residue. The residue at position 80 was then moved toward Ala-177 along an axis defined by the vector through their alpha carbon atoms, and every 0.25 Å, a conformation was saved using VMD1.8.4 (Humphrey et al. 1996). Each of these conformations was then used to seed two in vacuo molecular dynamics simulations of 10 ns duration where the positions of the alpha carbons were fixed. Simulations were also performed where the entire TM backbones were fixed, and similar results were obtained (data not shown). The hydrogen-bond propensity was measured using VMD as a function of the separation between the two amino acid residues from this range of simulations. The distribution of distances between the two residues was also computed from these trajectories. All simulations were performed using NAMD2 (Kalé et al., 1999; Phillips et al., 2005) and were analyzed using VMD. The CHARMM27 forcefield (MacKerell et al., 1998), with explicit hydrogens, was used and all residues were kept in their default ionization states. A hydrogen bond is assumed to have formed if the distance between the donor and acceptor heavy atoms is <3.5 Å and the angular deviation is <30°. At each separation distance, 200 steps of energy minimization were performed followed by 10 ns of molecular dynamics. The first 2.5 ns were discarded from all calculations to remove any transient effects. No explicit waters were included, and SHAKE (Miyamoto and Kollman, 1992) was used to allow an integration time step of 2 fs. A Langevin thermostat was applied to maintain the temperature at 310 K. Periodic boundary conditions were not used, and van der Waals and electrostatics were both calculated using a cutoff of 17 Å with a switching function applied from 15 Å.

### Making the Open-State Model of Kir1.1

The key structural features of the homology model of the closed state of Kir1.1 were separately fitted onto the existing open-state model of the transmembrane helices of KirBac3.1 (Kuo et al., 2005) in several stages. First, the sequences of Kir1.1 and KirBac3.1 were aligned. Then the inner, outer, and slide helices of the closed Kir1.1 model were structurally fitted onto the open-state model of KirBac3.1 (the inner TM2 helix was divided into two at the glycine hinge point). This generated an open-state model of the TM helices of Kir1.1 that closely resembled that of KirBac3.1. To help make clear the differences between the two states a movie was made by linearly interpolating between the two states (see Supplemental Data). This is only intended to assist with a visualization of the types of motions that are likely to occur. The pictures and the movie were rendered using VMD (Humphrey et al. 1996) which was also used to structurally align the proteins.

## Supplemental Data

Document S1. Three Figures

Movie S1. How Kir1.1 Might Move between the Open and Closed States This movie was produced by linearly tweening between the open and closed states using VMD. It illustrates the types of motions necessary to open the channel and the possible role of Lys-80 and Ala-177 in stabilizing the closed state. The view is from beneath the channel up through the pore. TM1, TM2, and the slide helix are shown in green, yellow, and red, respectively. The two amino acid residues (Lys-80 and Ala-177) are drawn as tubes and are colored according to element.
