# Supplementary material for: H Bonding at the Helix-Bundle Crossing Controls Gating in Kir Potassium Channels
Source: Neuron. 2007 Aug 16;55(4):602–14. doi: 10.1016/j.neuron.2007.07.026 (PMC1950231; doi:10.1016/j.neuron.2007.07.026)
Supplement: Document S1. Three Figures [file mmc1.pdf]

Neuron, Volume *55*

Supplemental Data

H Bonding at the Helix-Bundle Crossing

Controls Gating in Kir Potassium Channels

Markus Rapedius, Philip W. Fowler, Lijun Shang, Mark S.P. Sansom, Stephen J. Tucker, and Thomas Baukrowitz

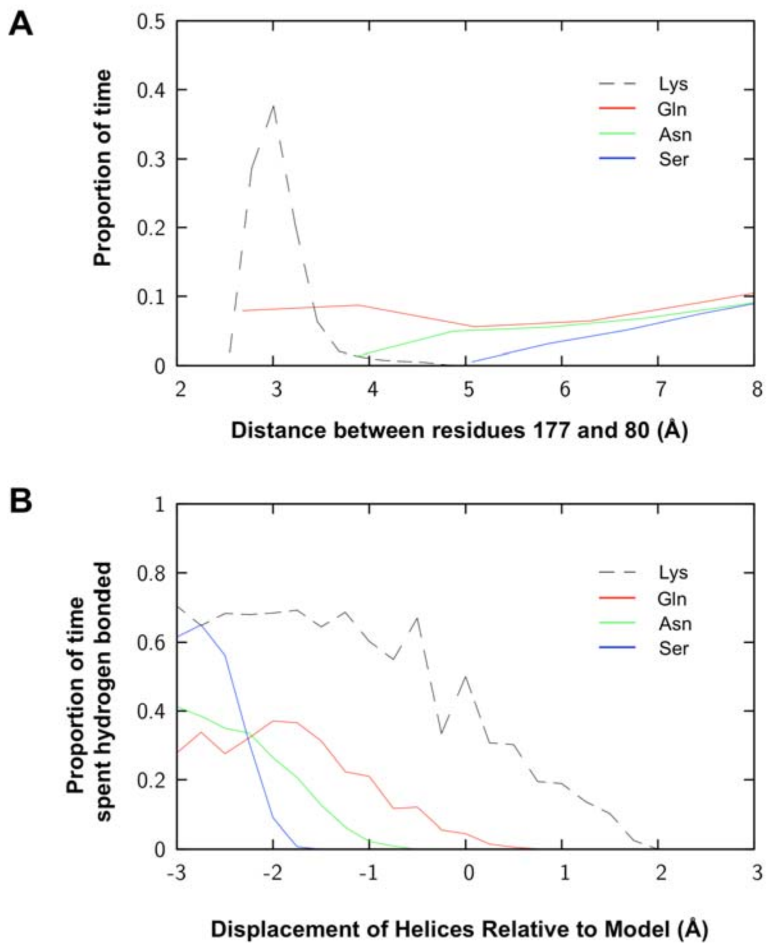

Figure S1. MD Simulations of Interactions between TM1-TM2

A. The distribution of distances between the carbonyl oxygen of Ala177 and the side-chain heavy atom of X80, where X is either Lys, Gln, Asn or Ser. This was calculated from the molecular dynamics simulations (see Experimental Procedures) where the separation between TM1 and TM2 was fixed at the distance found in the closed state homology model. It can be clearly seen that only Lys and Gln approach closer than 4 Å. This indicates that it is likely that a hydrogen bond has formed between Lys80 and Ala177 and possible that a hydrogen bond is able to form between Gln80 and Ala177.

B. This is confirmed by examining how the hydrogen bond propensity varies as the distance between Ala177 and X80 is altered. At zero displacement, corresponding to the same separation in the closed state homology model, Lys80 and Gln80 spend 50% and 5% of the time, respectively, hydrogen bonded to Ala177. For hydrogen bonds to form between Ala177 and Asn80 or Ser80, the helices must be brought at least 1-2 Å closer than predicted by the Kir1.1 homology model.

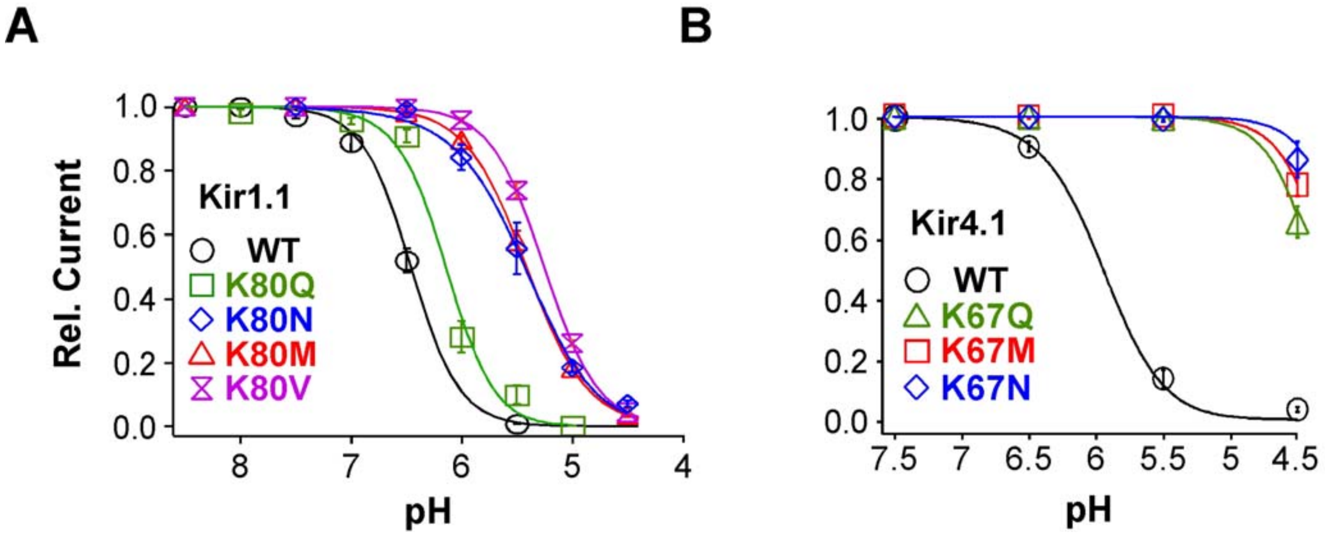

Figure S2. Mutants at the TM1 Site Change pH Sensitivity in Kir1.1 and Kir4.1 Channels

A. Dose-response curves for pH inhibition for Kir1.1 and Kir4.1 (**B.**) with indicated mutants. Data points represent mean ± SEM of at least five individual experiments. Curves were fitted to standard Hill equation and the corresponding IC_{50} fit values are plotted in Fig. 5. To obtain an estimation of the IC_{50} values in Kir4.1-K67N/M/Q the data were fitted to a Hill equation with a constrained Hill coefficient (n = 2.6, similar to the Hill coefficient of WT-Kir4.1, n = 2.6 ± 0.2).

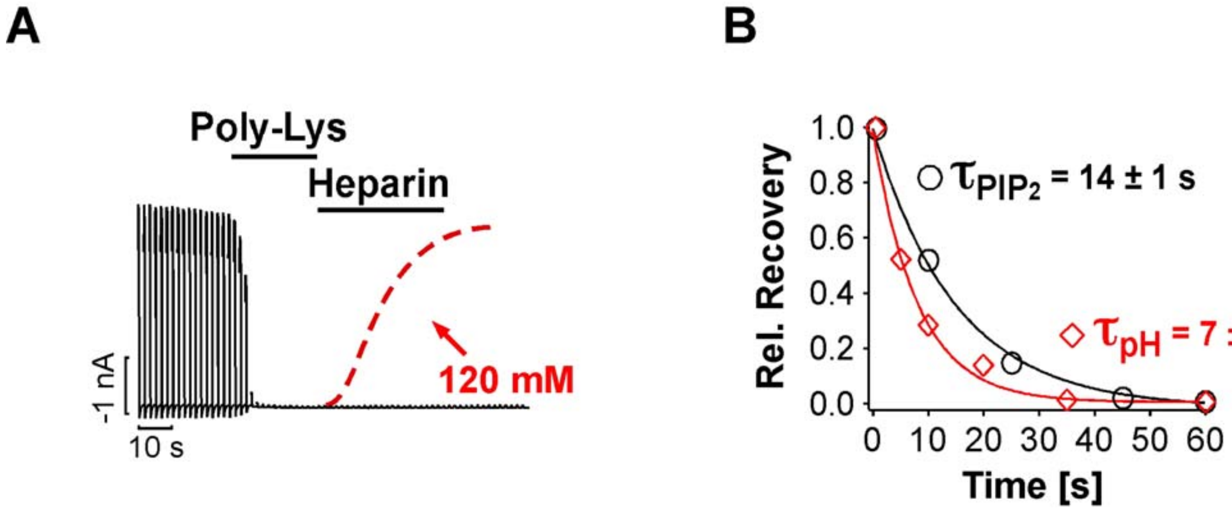

Figure S3. Poly-Lysine and pH-Induced K^+-Dependent Inactivation in Kir1.1 Channels

A. Inhibition of Kir1.1 channels by poly-lysine (250 µg/ml) and lack of reactivation by heparin (to remove poly-lysine) in the absence of extracellular K^+ (120 mM NMG^+ solution); red dotted line represents the time course of heparin reactivation typically obtained in the presence of 120 mM K^+ extracellular.

B. Time course of channel inactivation in the absence of extracellular K^+ (120 mM NMG^+ solution) as monitored by the current recovery (by heparin) subsequent to increasing time intervals of complete channel inhibition by poly-lysine (black line) and current recovery upon alkalization (pH 8.0) subsequent to increasing time intervals of complete channel inhibition by pH 6.0 (red line); τ values represent mean ± SEM, n = 3.
